# Assessment of knowledge of use of electronic cigarette and its harmful effects among young adults

**DOI:** 10.1515/med-2020-0224

**Published:** 2020-08-25

**Authors:** Vidushi Gupta, Madhu Sharma, Natarajan Srikant, Nidhi Manaktala

**Affiliations:** Interns, Department of Oral Pathology and Microbiology, Manipal College of Dental Sciences, Mangalore, Manipal Academy of Higher Education, Karnataka, India; Department of Oral Pathology and Microbiology, Manipal College of Dental Sciences, Mangalore, Manipal Academy of Higher Education, Karnataka, India; Department of Oral Pathology and Microbiology, Manipal College of Dental Sciences, Mangalore, Manipal Academy of Higher Education, Karnataka, India

**Keywords:** vaping, electronic nicotine delivery system, e-cigarette, harmful effects young adults

## Abstract

**Background:**

The practice of young adults smoking e-cigarette may have been adopted as a way of smoking cessation or just to follow a trend. Most people still remain unaware of the detrimental effects of e-cigarette. This study was carried out to assess the prevalence of the habit of smoking e-cigarette and the awareness of its harmful effects among adults aged 18–23 years.

**Methods:**

A questionnaire adopted from a study conducted in Italy was used in the study following due permission. After taking their consent, participants were requested to fill the survey irrespective of their smoking status.

**Results:**

In total the study comprised 710 participants aged 20.7 ± 1.7 years (females = 412 and males = 298) from six countries. Most respondents were from India followed by the USA and then the UK. The most common mode of information for the participants on the existence of e-cigarette was from the family members, friends followed by Internet search engines and TV/newspaper advertisements. Among the participants, e-cigarette had a prevalence of 5.63%. Among these, 26 participants were using e-cigarette for less than 1 year and 2 participants for more than 5 years. E-cigarette smokers also experienced sore throat, cough, headache, dizziness and sleeplessness.

**Conclusion:**

Majority of the young population was unaware about the use and harmful effects of e-cigarette.

## 1 Introduction

The number and popularity of the electronic nicotine delivery systems, especially e-cigarette, have been increasing in the last decade. Although these systems owe their popularity to the exclusion of the harmful chemicals that are present in tobacco smoke, there is a debate whether they are safe, regulated, as harmless as they are assumed to be and have potential unknown long-term effects [1]. E-cigarette smoking is becoming a fast-growing trend among young adults. The handheld electronic devices, commonly known as electronic cigarettes or e-cigarette, are gaining wide acceptance as they do not cause bad breath and have no flame and carbon monoxide emission, as opposed to the conventional tobacco cigarettes. The lack of lighting/fire that prevents staining of teeth, fingers or nails. The availability in different flavours and the lesser known side effects have contributed to its increasing popularity. It can be consumed as a vapour (vaping, inhalation of aerosols) or an atomizer (heating element that atomizes the e-liquid) [2].

Although the early concept of e-cigarette appeared in a patent developed by H. A. Gilbert in 1963, the modern day e-cigarette was invented by a Chinese pharmacist Hon Lik in 2003, who introduced it to the market the following year. His design was intended to create a smoke-like vapour to be an alternative to smoking. The Ruyan E-cigar was the first ever E-cigarette launched in China in 2004 and introduced to the Chinese domestic market in 2004 [3]. E-cigarette came in the US and the European markets in 2006 and 2007, respectively. The company that Lik worked for, Golden Dragon Holdings, registered an international patent in November 2007. In recent days, most e-cigarette uses a battery-powered heating element, rather than the earlier ones that used the ultrasonic technology [4].

A lot has been described about the contents of e-cigarette. An e-cigarette consists of an e-liquid which contains five main ingredients – vegetable glycerine, propylene glycol, flavour, nicotine and distilled water along with the potential carcinogens like formaldehyde [5]. The 250+ components that are found in this e-liquid also have been found to cause numerous detrimental effects. Some of these include periodontal and pulmonary diseases, gingivitis, asthma, allergies, insomnia, anxiety, depression, chest pain, various heart ailments and possible damage to the oral mucosa.

The prevalence of e-cigarette use is relatively low in India compared to other well-developed countries like the USA or the UK. Taking this into consideration, it will be better for medical professionals to be well versed with the new trends and their risk potential [6].

There has been an injudicious use of e-cigarette, and the general population using it has no knowledge about the long-term consequences of using e-cigarette. Simultaneously, many people are unaware about the existence of the same, and hence the present study was conducted to assess the awareness about the use and harmful effects of E-cigarette smoking among young adults (aged 18–23 years). The study also aimed to assess the attitude of young adults towards e-cigarette smoking.

## 2 Materials and methods

The study included 710 participants, including smokers and non-smokers across seven countries (India, USA, Canada, Australia, UK, Africa and South America), from varying socio-economic status. Young adults in the age group of 18–23 years were included in the study sample.

**Figure 1:**
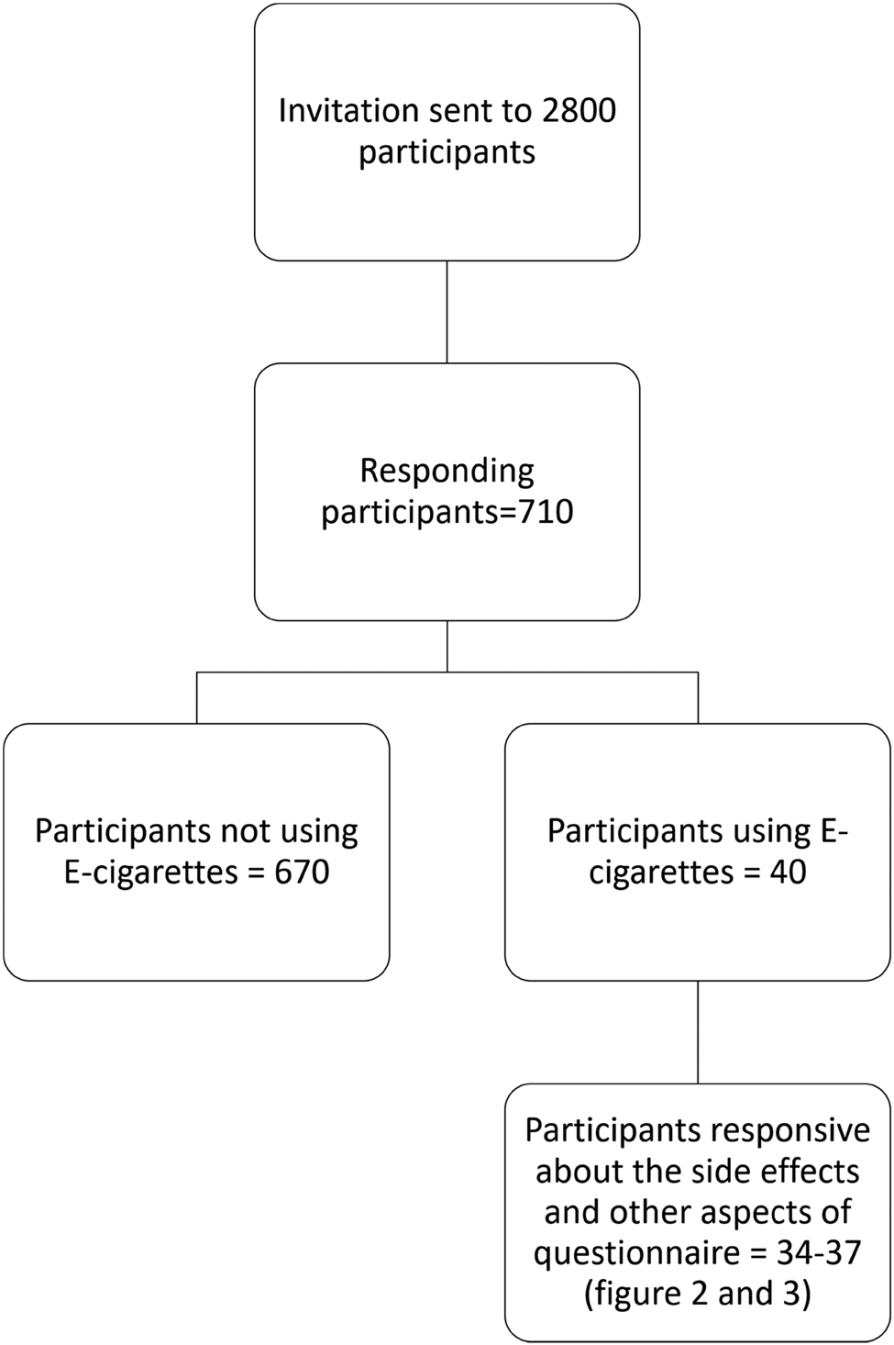
The consort diagram of study participants.

Figure 1 presents the details of the response rate and the final number of participants included in the survey.

Before the commencement of the study, clearance from the institutional ethics committee was obtained, and the consent obtained from each person was documented to ensure their voluntary and active participation in the study. In addition, the conducted research was not related to human or animal use. The overall sample size was calculated using the following formula:

n={\left(\frac{{Z}_{\alpha /2}}{d}\right)}^{2}p(1-p)

On the basis of the article titled “Characteristics, perceived side effects and benefits of e-cigarette use: a world-wide survey of more than 19,000 consumers” [2], the proportion of side effects observed was 59.8%. With an alpha of 5%, the corresponding *Z* value being −1.96, using the above-mentioned formula keeping the minimum percentage difference to be deemed clinically significant as 3.8%, the sample size required was calculated to be 640 for the study.

The questionnaire used in this study was adopted from Farsalinos et al. [2] after obtaining permission from the authors but modified thereafter. From the original questionnaire, a few questions were omitted or modified, including the height and weight of the patient being omitted and the highest level of education was not asked as most of them were young adolescents. Additionally, to remove any bias, the origin/source of the survey was not addressed in the questionnaire. Questions related to the habit of tobacco cigarette use along with e-cigarette (Q12) and awareness regarding the e-cigarette liquid (Q15) were added and modified.

The modified questionnaire (Annexure 1) was prepared in the Google form platform, and data were collected during a 6-month period. The Google-form-based survey was circulated directly as well as uploaded on various online survey portals like SurveyMonkey and SurveyPlanet, for the participants in the respective age group. It was also uploaded on various social media platforms like Facebook and Twitter, requesting volunteers to participate. Among the demographic questions, nationality was included to identify the country of origin of the participant. The data obtained from the study were compiled and statistically analysed using the statistical package for social sciences (SPSS) software version 20.0 and studied using frequency distribution. Upon completion and submission of the questionnaire, relevant educational material regarding the harmful effects of E-cigarette use was sent to the volunteers via email.

## 3 Results

### 3.1 Demographic profile

Around 400 participants from each continent were targeted, leading to a sample size of almost 2,800. However, only 710 (25.35%) participants responded to the questionnaire.

The study participants were predominantly from India (*n* = 602), and participants from other countries included the USA (*n* = 48), the UK (*n* = 10), Australia (*n* = 2), Canada (*n* = 9), South America (*n* = 34; Peru = 6, Argentina = 16, Chile = 5 and Brazil = 7) and Africa (*n* = 5; Cameroon = 1, Tanzania = 1, Egypt = 1 and South Africa = 2). Majority of the individuals were females (58%, *n* = 412) in the age range of 18–23 years (mean age of 20.65 ± 1.7 years).

### 3.2 General awareness

All had knowledge about e-cigarette, and majority of them got to know about them through the family or friends (36.8%) followed by Internet search engines (20.5%), TV/radio/newspaper (17.6%) and websites (11.7%). Majority of the individuals (of the 710 individuals) were regular smokers <1 year (mean of 0.874 years) and used 1–2 cigarettes per day. The mean age of the participants was 20.65 ± 1.7 years. An average of 1.57 ± 16.56 cigarettes were smoked among the 710 interviewed individuals with an average duration of 0.874 ± 4.69 years.

### 3.3 E-cigarette usage profile

Among the 710 individuals, 40 were using e-cigarette, with a prevalence of 5.63%, and among them majority were using it for <1 year (*n* = 26, 65%). Among the 40 e-cigarette users, 18 were from India, 10 from the USA, 3 from the UK, 8 from South America and 1 from Africa. The analysis of the use of liquid among the users (*n* = 40) showed that 41% (*n* = 16) used prefilled cartridges, 35.9% (*n* = 14) used bottles with ready-to-use liquid and 9 (23%) used liquid base with flavours. Majority of the individuals (38.5%) did not have the knowledge of the quantity of the liquid they consumed. Approximately 25.6% individuals used 1–2 mL of fluid per day.

Before starting the use of e-cigarette, the e-cigarette users had asthma (*n* = 4), three individuals had diabetes, hypertension and lung diseases like emphysema, chronic bronchitis and chronic obstructive pulmonary disease (COPD); two individuals had high cholesterol, thyroid gland dysfunction (hypothyroidism or hyperthyroidism) and coronary heart conditions. Majority of these individuals (*n* = 6) reported that the pre-existing diseases that they had did not worsen after the initiation of the habit. Interestingly, two individuals claimed that the symptoms got better following the initiation of e-cigarette.

With respect to the reason for the initiation of the habit, majority (14, 36.8%) started the habit to quit or reduce smoking followed by a desire to enjoy the variety of flavours offered by e-cigarette (*n* = 8, 21.1%). Prior to the use of e-cigarette, individuals had tried nicotine replacement therapy (21.1%), smokeless tobacco (13.2%), oral medications (5.3%) and/or psychological counselling (2.6%) to quit smoking. Majority of the individuals (66.6%) considered e-cigarette to be less harmful than tobacco cigarettes (Table 1).

**Table 1:** Awareness of e-cigarette usage and effects in 38 cases using frequency distribution (questions modified from Farsalinos et al. [2])

		Frequency (%) *n* = 38
Reason for starting e-cigarette	Avoid smoking ban in public places	1 (2.6)
	Enjoy a variety of flavours that only e-cigarette offers	8 (21.1)
	I had already quit smoking, but I wanted to avoid relapse (restart smoking)	7 (18.4)
	Just to give it a try	4 (10.5)
	Quit or reduce smoking and reduce smoking exposure of family members	14 (36.8)
	Spare money	4 (10.5)
Do you think e-cigarette is	Absolutely healthy and safe	5 (12.8)
	Equally harmful to tobacco cigarettes	4 (10.3)
	Less harmful than tobacco cigarettes	26 (66.7)
	More harmful than tobacco cigarettes	4 (10.3)
Methods used in the past to quit smoking	Nicotine replacement therapy (patch, gums and other nicotine products)	8 (21.1)
	Oral medications (pills) approved for smoking cessation	2 (5.3)
	Psychological counselling and support	1 (2.6)
	Self-motivation	13 (34.2)
	Smokeless tobacco (sinus, dissolvable tobacco, chewable tobacco, etc.)	5 (13.2)
	Others	9 (23.7)
If e-cigarette or liquids with nicotine levels you use were banned from the market, you would:	Search for e-cigarette or nicotine-containing liquids in the black market or other sources (even illegal sources)	4 (10.5)
	Start smoking again	13 (34.2)
	Start smoking again; search for e-cigarette or nicotine-containing liquids in the black market or other sources (even illegal sources)	1 (2.6)
	Stop using e-cigarette without starting to smoke again	20 (52.6)
What did your physician advice you concerning the use of e-cigarette?	Did not express any opinion	4 (10.8)
	To continue using it if it helps me stay off or reduce smoking	5 (13.5)
	To stop using it or never use it	4 (10.8)
	You did not inform your physician	24 (64.9)
During the whole period of using the e-cigarette, did you ever completely quit smoking but later relapsed and started smoking tobacco cigarettes again?	Yes	11 (28.9)
Have you ever used the e-cigarette device to inhale substances other than liquids or prefilled cartridges specifically made for it?	Yes	8 (21.1)
How do you usually measure your daily e-cigarette consumption? By measuring	The number of millilitres of the liquid consumed per day	15 (40.5)
	The number of prefilled cartridges you use per day	9 (24.3)
	The number of puffs per day	7 (18.9)
	The number of times you use the e-cigarette device per day	6 (16.2)
	EGO-type batteries “Mods” (variable voltage/variable wattage devices and/or custom atomizers)	21 (55.3)
What kind of an e-cigarette device do you most often use?	Small devices that look similar to tobacco cigarettes	17 (44.7)

Before the start of the habit of vaping, 42.1% individuals felt it difficult to refrain from smoking in banned areas; majority (47.1%) had the habit of smoking e-cigarette within the first hour of waking up, and 39.5% used to smoke even when they were unwell (Table 1).

### 3.4 Attitude of E-cigarette smokers

The analysis of the habit of E-cigarette use demonstrated that 35.1% of the individuals were unwilling to give up the smoking habit, while 34.2% people felt that they would start smoking tobacco cigarettes if e-cigarette was to be banned. Surprisingly, among the 13 individuals who did inform the physician about their vaping habit, 5 were advised to continue using e-cigarette if it helps them stay off or reduce smoking. During the period of e-cigarette use, 71.1% of the individuals (E-cigarette users) still continued to use the regular tobacco cigarettes. The individuals also inhaled substances other than the prefilled cartridges, specifically made for it, in their E-cigarette instrument (21.1%; Table 1).

Majority (40.5%) kept tabs on their usage of e-cigarette by measuring the amount of liquid consumed in the cartridge. The type of e-cigarette used was either powered by EGO-type batteries (55.3%) or small devices, which look similar to tobacco cigarettes (44.7%; Table 1).

### 3.5 Harmful effects of e-cigarette

Immediately after starting the habit of e-cigarette, the individuals were reported to have sore throat, dizziness, headache, cough, chest pain and dry mouth, which partly resolved in 22.2% individuals. Apart from the individual who smokes e-cigarette, the associated contact persons (12.8%) also had health problems. Two cases of battery explosion and three cases of electrical shock were documented among participants using e-cigarette (Table 1).

**Figure 2:**
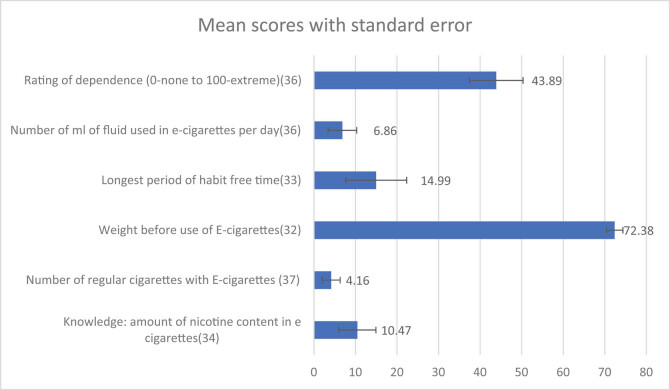
The mean scores of age and usage details of e-cigarette habits.

The amount of nicotine content used per day was variable, ranging 10.46 ± 26.04 mL (with an average use of 6.86 mL), and the number of regular cigarettes smoked increased to an average of 4.16 ± 13.1 cigarettes per day. Before the use of e-cigarette, on an average 14.9 months (range 0–240 months), individuals remained free from smoking. On an average, 43.89% was the dependence score of the individuals (Figure 2).

**Figure 3:**
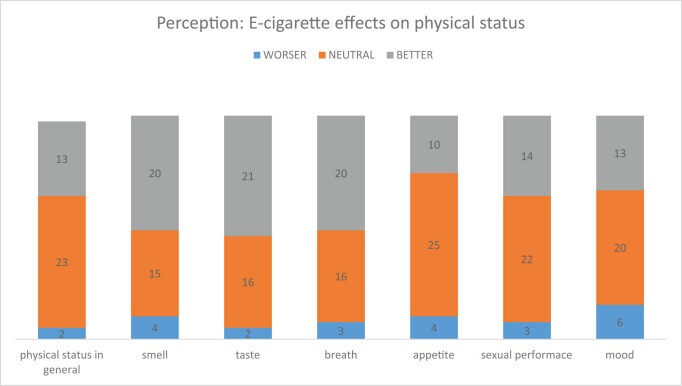
The perception of the physical status after the use of e-cigarette.

Following the use of e-cigarette, the users report betterment of physical status, smell, taste, breath, appetite, sexual performance and mood to varying degrees. However, a small percentage did report worsening of these features (Figure 3).

## 4 Discussion

E-cigarette is a small battery-operated handheld electronic device and is commonly known as e-cigarette. The history of e-cigarette can be traced to Herbert A. Gilbert who patented it in 1963 as “a smokeless non-tobacco cigarette.” It was not much popular then and was not commercialized [3].

Our study aimed to assess the knowledge and awareness regarding e-cigarette usage, and the results showed a reduced awareness of the side effects of the use of e-cigarette. Several myths associated with e-cigarette smoking need to be busted with ground realities. Around 79.5% (31 of 39) of the participants felt that e-cigarette is safer and healthier (*n* = 5, 12.8%) and rather less harmful (26, 66.7%) than the conventional cigarettes. However, that is not the case considering the harmful effects related to these in the long run. E-cigarette is gaining wide acceptance, especially among young adults. The practice of young adults smoking e-cigarette may have been adopted as a way of smoking cessation or just to follow a trend. They are used as a method of reducing tobacco smoking, as they do not cause bad breath, have no flame and carbon monoxide emissions, as opposed to the conventional tobacco cigarettes. They do not cause staining of teeth, fingers or nails. The lesser known side effects have contributed to its increasing popularity. However, most people remain unaware of the damaging ramifications of smoking e-cigarette.

E-cigarette liquids are available in prefilled cartridges with different amounts of nicotine. As seen in the study, most of the participants remained ignorant of the amount of e-cigarette liquid they consumed. Though a good number of the participants had an idea about the amount of e-liquid they use, the amount of nicotine they consumed was unknown; a few respondents also admitted to using the vape to smoke and inhale other substances. In such cases, damage control can be done by limiting the use of nicotine intake by the users. E-cigarette has cartridges with the liquid containing more than 250 components, and the main components being vegetable glycerine, propylene glycol, flavour, nicotine and distilled water in addition to potential carcinogens like formaldehyde. These are vapourized by the battery upon inhalation and imitate real cigarette smoking [5]. These vapour cigarettes have been readily available only since 2006. Hence, there is limited research on its ill effects. E-cigarette was initially popularised as a safer way to intake nicotine, but doctors still do not approve of it as a form of smoking cessation. These flavoured e-cigarette and cartridges contain chemicals like dactyl 2, 3-pentanedione and acetone which are known to cause bronchiolitis obliterates or popcorn lung when heated, vapourized or inhaled. The formaldehyde component can cause brain cancer, chronic myeloid leukaemia and nasopharyngeal cancer. It does not contain anabasine, nornicotine and anatabine, the lack of which makes tobacco cigarettes more addictive. Various popular flavours are produced due to the addition of benzaldehyde, which can cause respiratory tract inflammation. They also contain metals like cadmium, chromium, lead, manganese and nickel, which can cause brain damage, respiratory problems and cancer after chronic usage. Some liquids contain arsenic, which is highly toxic, and may lead to liver diseases and even permanent brain damage/coma [7].

One of the components of e-cigarette, an anti-freeze component called diethylene glycol, is toxic and can cause respiratory problems in humans. Low levels of cadmium, another toxic metal found in the vapours, is known to cause breathing problems. Tetramethylpyrazine can result in brain damage. In some e-liquids, the liquid nicotine is substituted with liquid tetrahydrocannabinol (THC). THC is a potent marijuana resin that can cause addiction and pose a threat to health. Cancer-causing compounds like nitrosamines are also present in this e-liquid. These digital smokes also cause allergies and asthma, various heart diseases, heart attacks and other ailments. Mood disorders and impulse control failure may be a threat. Other side effects include periodontal and pulmonary diseases, gingivitis, insomnia, anxiety, depression, chest pain and possible damage to the oral mucosa. Nicotine poisoning may cause burning sensation in mouth, drooling or increased saliva, fainting or coma, convulsions, headache, muscle twitching, vomiting, weakness, agitation, restlessness, excitement, confusion and palpitations. If overheated, the battery might explode causing tooth loss, soft tissue loss and injuries to face, hands, thighs and groin [8,9].

On assessing the changes in the physical status after starting vaping, it was found that most of the participants were neutral. However, a small proportion experienced exacerbation of conditions like alterations in taste, breath, mood, appetite, smell and sexual performance. The physical status had also deteriorated with effects like oral inflammation, respiratory symptoms, cardiac symptoms and allergic reactions. Mishaps while vaping were also found to be common. The accidental consumption of e-liquid and battery explosion lead to burns around the mouth. Electrical shocks from the charger and the device were also experienced by some.

According to other researches (Farsalinos et al.) [2], e-cigarette has been used as long-term tobacco cigarette substitutes. Mild temporary side effects were reported by the participants. However, according to the present study, a few of the digital smokers experienced various ill effects, from which most were unresolved.

The use of e-cigarette is associated with greater risk if there is subsequent tobacco cigarette smoking initiation; however, a strong e-cigarette smoking regulation could possible ledge the use of tobacco cigarettes among the youth as stated in a study by Soneji et al. [10].

Other previous studies indicate that the participants were well aware of the harmful effects of smoking cigarette and considered e-cigarette less harmful than the conventional cigarettes [11,12]. In our study, it was found that 66.7% (26 of 39) of the participants considered e-cigarette to be less harmful and 12.8% (5 of 39) participants considered e-cigarette absolutely safe.

On the positive side e-cigarette has less carcinogens compared to tobacco cigarettes, thus posing to be less cancerous. However, the fact that it damages more systems in the body leading to equal damage, if not more than conventional cigarette, cannot be ignored. E-cigarette not only has a social impact but also has environmental consequences [11]. E-cigarette, in addition to helping with nicotine addiction, causes other harms, especially to the respiratory system, and the chemical exposures lead to numerous health consequences. Such potential risks need to be highlighted more to create an awareness about the negative side of the sword [12].

A study by Gravely et al. [13] also reported the awareness regarding the Alternate Nicotine Delivery System (ANDS). In their study, the awareness of e-cigarette ranged from 88% in the Netherlands to 34% in China. The trial of e-cigarette ranged from 20% in Australia to 2% in China. The current use of e-cigarette ranged from 14% in Malaysia to 0.05% in China. They found that there was considerable cross-country variation in both awareness and the use of e-cigarette [13]. In addition, Polosa et al. considered the use of E-cigarette as a double-edged sword as, according to them, one should not lose sight of the potential benefits of e-cigarette compared to the conventional tobacco cigarettes as most people still smoke conventional cigarettes, and this will be a public health issue for a number of years to come [14].

The study’s main limitation was the selection bias. The participants were recruited by self-volunteering over the social network, leading to no proper representation taking place for each country/continent. In addition, the response rate was poor, accounting for only 25% of the actual target. This may lead to the inability to generalize the findings in a population.

However, on the upside, this study is the first of its kind in India, with the main aim being spreading awareness among young adults about the harmful effects of this fast-growing trend as the population overdosing on e-cigarette has increased. In the interest of public health, the awareness should be spread among the public about the harmful effects of e-cigarette smoking. However, additional research and longitudinal studies are needed to understand the patterns and impact of global ANDS use in greater detail, particularly on the impact of e-cigarette on dual use, quitting, relapse among smokers, and the initiation of smoking among youth. There is a need to address the critical question of whether e-cigarette represents a positive, negative or mixed phenomenon for tobacco control and public health. Studies that would be able to measure and understand the interplay between the ANDS and the combustible tobacco products should be conducted, published and brought to the attention of policy-makers to make evidence-based judgements about the net burden of ANDS on the tobacco pandemic, within and across countries.

**Figure 4:**
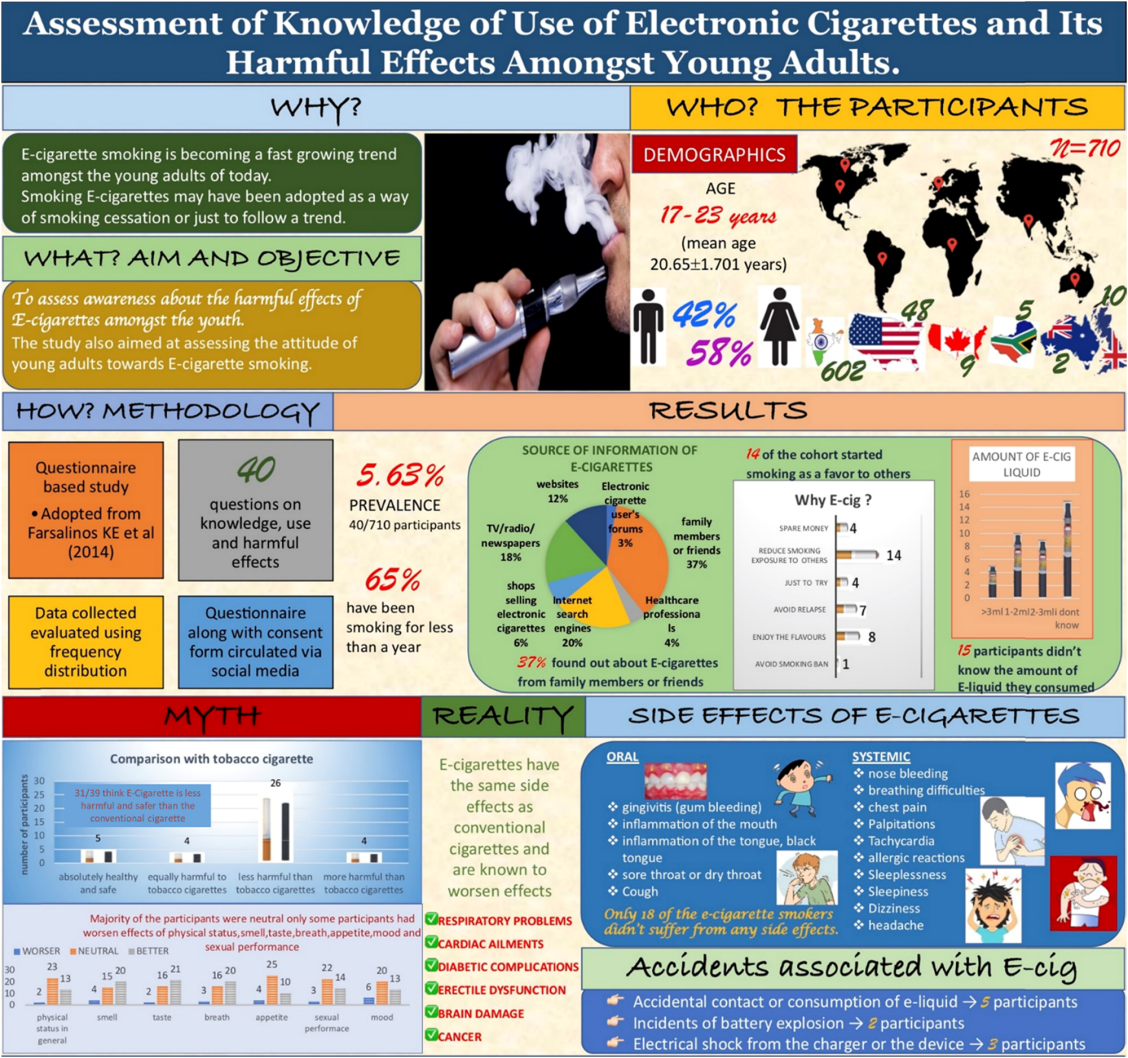
A pictorial representation of a detailed summary of the study.

A detailed summary of the study is depicted in Figure 4.
